# Unincreased risk of hospitalized infection under targeted therapies versus methotrexate in elderly patients with rheumatoid arthritis: a retrospective cohort study

**DOI:** 10.1186/s13075-022-02807-9

**Published:** 2022-06-10

**Authors:** Ryoko Sakai, Eiichi Tanaka, Masako Majima, Masayoshi Harigai

**Affiliations:** 1 Division of Rheumatology, Department of Internal Medicine, Tokyo Women’s Medical University School of Medicine, 8-1, Kawada-cho, Sinjuku-ku, Tokyo, 162–8666 Japan; 2 Division of Multidisciplinary Management of Rheumatic Diseases, Department of Rheumatology, Tokyo Women’s Medical University School of Medicine, 8-1, Kawada-cho, Sinjuku-ku, Tokyo, 162–8666 Japan

**Keywords:** Biological DMARD, Elderly, Infection, Janus kinase inhibitor, Rheumatoid arthritis, Risk, Targeted therapy

## Abstract

**Background:**

Infection is one of the primary concerns during treatment for rheumatoid arthritis (RA) in elderly patients. However, infection risk of patients with RA receiving targeted therapy (TT) including biological disease-modifying antirheumatic drugs (bDMARDs) and Janus kinase inhibitors (JAKIs) in elderly patients are scarce. The aim of this study was to compare the risk of hospitalized infection (HI) with TT versus methotrexate (MTX) therapy among young, elderly, and older elderly patients with RA.

**Methods:**

Using Japanese claims data, patients satisfying the following criteria were enrolled: (1) ≥ one ICD10 code for RA; (2) ≥ one prescription of MTX or TT (bDMARDs and JAKIs) between April 2008 and September 2018; and (3) ≥16 years old. We calculated the incidence rate (IR) of HI per 100 patient-years in the young, elderly, and older elderly groups (those aged 16–64, 65–74, and ≥75 years, respectively) and the IR ratio (TT vs. MTX) of HI. A logistic regression model was used to estimate the associations between HI and TT versus MTX in each group.

**Results:**

The overall IR of HI per 100 patient-years (95% confidence interval) was 3.2 [2.9–3.5], 5.0 [4.6–5.4], and 10.1 [9.5–10.9] in the young, elderly, and older elderly groups, respectively. Concomitant use of MTX or immunosuppressive DMARDs with TT was less frequent in the elderly and older elderly groups. The adjusted odds ratio of TT vs. MTX for HI was 1.3 (1.0–1.7; *p* = 0.021), 0.79 (0.61–1.0; *p* = 0.084), and 0.73 (0.56–0.94; *p* = 0.015) in the young, elderly, and older elderly groups, respectively.

**Conclusion:**

The overall IR of HI was increased with age. The risk of HI under TT compared to MTX was not elevated in elderly and older elderly patients after adjusting for patients’ characteristics and concomitant treatments.

**Supplementary Information:**

The online version contains supplementary material available at 10.1186/s13075-022-02807-9.

## Background

The number of elderly patients with rheumatoid arthritis (RA) has recently increased due to improved vital prognosis of patients with RA [1], increased incidence of elderly onset RA [2, 3], and higher cumulative risk of RA in elderly women [4]. In Japan, one of the countries with the highest aged society in the world, the proportion of patients with RA who are aged ≥ 65 years was 60.8%, and the largest proportion was 28.6% of patients with RA aged 70–79 years old [5]. Thus, the importance of safety data of elderly patients with RA has increased in clinical settings.

Generally, in elderly patients, it is difficult to apply the same treatment strategy as that used for non-elderly patients; this is due to the increase in morbidities, comorbidities, and treatment-related risks compared to those in younger patients [6]. Infection is one of the primary concerns during the treatment of RA, and previous studies have reported that older age is a significant risk factor for serious infections [7–10]. In addition to older age, medications for RA, such as biological disease-modifying antirheumatic drugs (bDMARDs) and high doses of oral corticosteroids (CS), have also been identified as significant risk factors for infection in patients with RA [7, 11–13]. Furthermore, it has been reported that the incidence rates of infection in clinical trials of Janus kinase inhibitors (JAKIs) were similar to those of control groups; however, these drugs also increased the risk of herpes zoster [14]. A recent study showed a different balance between the benefits and risks in elderly patients with RA; patients ≥75 years old under TNF inhibitor monotherapy had fewer discontinuations due to inefficacy than adverse events compared with younger patients with same treatment [15]. Thus, rheumatologists should seek for a better benefit–risk balance of immunosuppressive treatments for elderly patients with RA.

In elderly patients with RA who received bDMARDs, the most frequent serious adverse event was infection [8, 16], and elderly patients receiving bDMARDs had an approximately 1.6-fold higher risk of infection than young patients [17]. However, infection data of elderly patients with RA receiving targeted therapy (TT) (i.e., bDMARDs and JAKIs) are still limited. The purpose of this study was to compare the risks of hospitalized infection (HI) under TT among young, elderly, and older elderly patients with RA using the Japanese health insurance database.

## Methods

### Data source

We conducted a retrospective, longitudinal, population-based study using nationwide hospital-based claims data provided by the Medical Data Vision (MDV) Co., Ltd (Tokyo, Japan). The details of the MDV database have been described in our previous studies [18, 19]. In brief, more than 27.1 million patients who visited hospitals that participated in the Diagnostic Procedure Combination/per diem payment system (DPC/PDPS) in Japan (as of March 2019) were covered by the MDV database, which corresponded to 22% of the hospitals that participated in the DPC/PDPS. No personal identifiable information, such as patients’ names and addresses, is included in the data. The data include information on diagnoses, drug prescriptions, medical procedures, and reimbursement costs for hospitalization and outpatients.

### Study population

We enrolled the patients who met all of the following criteria: (1) at least one of the codes (M05.x; M06.x except for M061; or M08.x except for M081 and M082) from the International Statistical Classification of Diseases and Related Health Problems, 10th Revision (ICD-10); (2) at least one prescription of MTX, bDMARD, or JAKI between April 2008 and September 2018; and (3) age of 16 years or older. The above definition of cases with RA in the Japanese claims data was validated [20]. We excluded patients who were prescribed these drugs during the first 12 months in order to include only new users of MTX, bDMARDs, and JAKIs in the study population. In the new users of MTX and bDMARDs/JAKIs, we defined the first month of the prescription of these agents as the index month. Among the study population, we divided patients into three groups according to their age at the index month referring to the previous study [21]: young (16–64 years), elderly (65–74 years), and older elderly (≥75 years).

### Follow-up

The observation started from the index month and ended at 36 months, the last day of exposure to bDMARDs/JAKIs, the last day of exposure to MTX in patients who did not use bDMARD/JAKIs concomitantly, the month of loss of follow-up, or September 2019, whichever came first (Supplementary Fig. 1). The last day of exposure to MTX or JAKI was defined as the last day of a prescription of MTX or JAKI plus supply days and 30 days as a grace period. The last day of exposure to bDMARDs was defined as the last day of a prescription of bDMARDs, plus interval days of each agent and 30 days as a grace period [22]. We implemented an on-drug analysis for HI considering that the patients were censored at the end of exposure to MTX or bDMARDs/JAKIs with a 30-day grace period as described above.

### Definition of hospitalized infection

HI was defined by the ICD10 code for infection, with at least one prescription of antibiotic/antiviral/antifungal agents for each infection during hospitalization (Supplementary Table 1), considering the clinical setting in Japan. Some HIs were defined using the ICD10 code alone. In this database, no microbiological test or infection-related laboratory test results were available. In addition, we could not link this database with patients’ medical records or other databases; therefore, we could not distinguish infection that occurred outside of the hospital and those during hospitalization.

### Statistical analysis

The patients’ characteristics and the drugs used in each age group were described. We defined the patients’ comorbidities during the year before the index month. Patients with at least one ICD-10 code for diabetes mellitus (DM) (E10.x–E14.x) and at least one prescription for DM were defined as having comorbid DM. Charlson comorbidity index score *without age correction* was calculated, and other comorbidities such as chronic pulmonary disease and renal disease were considered to be present if patients had the corresponding ICD-10 codes based on coding algorithms for defining comorbidities [23]. Medication use for RA was defined during the index month. History of HI was defined using the definition of HI described above during the year before the index month. We described medication use during exposure to MTX or bDMARDs/JAKIs separately. We calculated the proportions of patients treated with each bDMARD/JAKI during exposure to bDMARDs/JAKIs and those who were prescribed immunosuppressive DMARDs (i.e., tacrolimus, mizoribine, and leflunomide) and oral CS during exposure to bDMARDs/JAKIs or MTX. The oral CS doses were converted to prednisolone equivalent doses. Fisher’s exact test was used to compare the proportion of patients among the three age groups.

The incidence rate (IR) per 100 patient-years (PY) and crude IR ratio (IRR vs. the young group) of HI during the observation period were calculated. Subsequently, in each age group, we calculated the IR per 100 PY of HI during exposure to MTX, bDMARDs/JAKIs, and IRR (TT vs. MTX). In addition, we compared the time until the month in which the first HI occurred among the age groups using the Kaplan–Meier method and log-rank test.

To estimate the associations between bDMARD/JAKI use and HI in each age group, we calculated the adjusted odds ratio (OR) of TT vs MTX exposure for HI using a logistic regression model. We used age, sex, comorbidity at the index month, medications for RA during the observation period (bDMARD/JAKI use, oral CS use), history of HI, and calendar year at the start of observation as covariates in the multivariable analysis considering medical importance. In the subgroup analysis, we prepared bDMARDs users group by excluding JAKI users from the bDMARD/JAKI users and calculated the OR of bDMARDs vs MTX exposure for HI in bDMARDs users group adjusting for the same variables using the logistic regression model. All statistical analyses were performed using SPSS version 23 (IBM Corp., Armonk, NY, USA).

## Results

### Study population

**Fig. 1:**
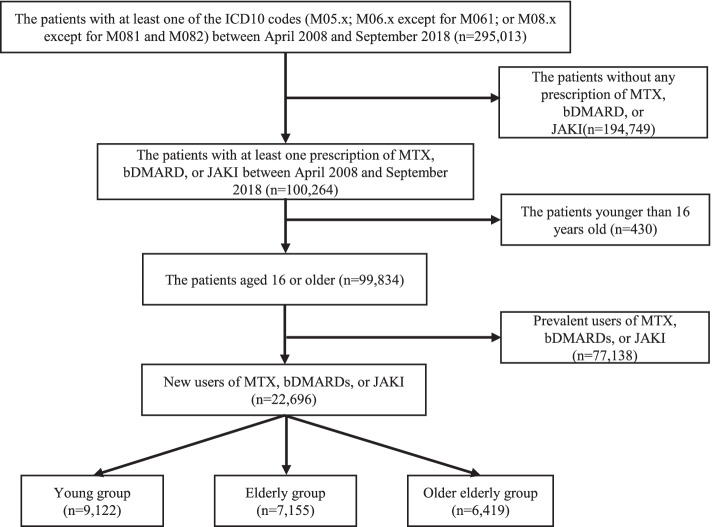
Patients’ flow. bDMARDs, biological disease-modifying antirheumatic drugs; ICD-10, the International Statistical Classification of Diseases and Related Health Problems, 10th Revision; JAKIs, Janus kinase inhibitors; MTX, methotrexate

The patients’ flow in this study was shown in Fig. 1. The baseline characteristics of the patients are shown in Table 1. The number of RA cases was 9122, 7155, and 6419 in the young, elderly, and older elderly groups, respectively. The median observation period (interquartile range [IQR]) was 26 months [13, 36] in the young group, 24 months [11, 36] in the elderly group, and 19 months [10, 34] in the older elderly group. More patients in the older and older elderly groups had comorbidities and a history of HI compared to those in the young group. In the young and elderly groups, over 80% of patients were prescribed MTX, and 76.9% of the patients in the older elderly group were prescribed MTX. The percentage of patients who were prescribed oral CS increased with the mean age. The median doses of oral CS in each group were similar.

**Table 1:** Patients’ characteristics at baseline

Variable^a^	Young group (16–64 years old, *n* = 9122)	Elderly group (65–74 years old, *n* = 7155)	Older elderly group (over 75 years old, *n* = 6419)
Median age [IQR]	54 [45, 60]	70 [67, 72]	79 [77, 83]
Sex, female, %	75.3	69.6	72.9
Median observation period, months [IQR]	26 [13, 36]	24 [13, 36]	19 [10,34]
Median Charlson Comorbidity Index score [IQR]	1 [1, 2]	2 [1,3]	2 [1,3]
Comorbidity, %			
Chronic pulmonary disease	12.4	16.2	17.2
Renal disease	1.8	3.5	4.9
Diabetes mellitus	6.1	11.4	11.0
History of HI	2.8	4.1	6.1
Medication use for RA, %			
MTX	83.0	82.3	76.9
bDMARDs	34.1	29.3	29.8
TNF inhibitor	22.3	15.6	14.5
Infliximab	4.0	1.6	0.6
Etanercept	6.6	5.2	5.3
Adalimumab	5.7	2.4	1.6
Golimumab	4.3	5.5	6.6
Certolizumab pegol	1.7	1.0	0.5
Tocilizumab	7.9	5.7	4.4
Abatacept	3.9	8.0	10.9
JAKIs	1.3	1.7	1.5
Tofacitinib	0.8	1.0	1.1
Baricitinib	0.5	0.7	0.4
Oral CS	43.5	47.8	55.5
Median dose of oral CS, mg/day [IQR]^b^	5 [4, 10]	5 [4, 7.5]	5 [4, 7.5]

*bDMARDs* Biological disease-modifying antirheumatic drugs, *CS* Corticosteroids, *HI* Hospitalized infection, *IQR* Interquartile range, *JAKIs* Janus kinase inhibitors, *MTX* Methotrexate, *RA* Rheumatoid arthritis, *TNF* Tumor necrosis factor

^a^Comorbidity and history of hospitalized infection were defined a year before the index month. Other variables were defined in the index month

^b^Doses of oral corticosteroids were converted to prednisolone equivalent doses

### Medication use during the observation period

Medication use in the young, elderly, and older elderly groups is shown in Table 2. During exposure to bDMARDs/JAKIs, the proportion of patients who were treated with TNF inhibitors in the older elderly group was significantly smaller than that in the young group (67.3% in the young group and 51.0% in the older elderly group; *p* < 0.001). Abatacept use was significantly more frequent in the elderly and older elderly groups than in the young group (14.4% in the young group, 27.8% in the elderly group, and 38.4% in the older elderly group; *p* < 0.001). A significantly lower proportion of patients in the elderly and older elderly groups were treated with MTX or immunosuppressive DMARDs under exposure to bDMARDs/JAKIs than in the young group (71.6% in the young group, 67.4% in the elderly group, and 54.7% in the older elderly group; *p* < 0.001). Moreover, the proportion of patients receiving concomitant MTX in the older elderly group was smaller than those in the other groups (63.0% in the young group, 52.9% in the elderly group, and 37.7% in the older elderly group; *p* < 0.001). Although the use of oral CS was more frequent in the elderly and older elderly groups than in the young group, similar proportions of patients were administered oral CS ≥10 mg/day of prednisolone across the three groups.

**Table 2:** Medication use during the observation period^a^

	Young group (16–64, *n* = 9122)	Elderly group (65–74, *n* = 7155)	Older elderly group (75–, *n* = 6419)	*P*-value^b^
During exposure to bDMARDs/JAKIs with or without methotrexate, %				
TNF inhibitors	67.3	56.6	51.0	< 0.001
Infliximab	12.6	6.1	2.6	< 0.001
Etanercept	20.6	18.0	17.2	0.001
Adalimumab	18.8	10.1	6.3	< 0.001
Golimumab	15.4	22.3	25.5	< 0.001
Certolizumab pegol	6.8	4.9	2.9	< 0.001
Tocilizumab	28.0	24.9	21.0	< 0.001
Abatacept	14.4	27.8	38.4	< 0.001
JAKIs	6.0	7.5	7.0	0.034
Tofacitinib	3.7	4.6	4.4	0.143
Baricitinib	2.3	3.1	2.9	0.140
Methotrexate or any immunosuppressive DMARD use	71.6	67.4	54.7	< 0.001
Methotrexate	63.0	52.9	37.7	< 0.001
Taclolimus	14.4	18.5	19.3	< 0.001
Mizoribine	2.3	3.6	3.8	< 0.001
Leflunomide	1.4	1.6	1.0	0.157
Oral CS use, %	52.4	57.4	66.2	< 0.001
Maximum dose of oral CS ^c^≥ 10 mg/day, %	19.5	18.6	21.7	0.017
Mean dose of oral CS ^c^≥ 10 mg/day, %	8.8	7.7	10.3	0.001
Mean dose of oral CS ^c^≥ 7.5 mg/day, %	11.6	12.0	15.5	< 0.001
During exposure to MTX without a bDMARDs and with or without other csDMARDs, %				
Any immunosuppressive DMARD use	12.7	10.9	10.2	< 0.001
Taclolimus	10.7	9.4	8.3	< 0.001
Mizoribine	1.8	1.6	2.0	0.354
Leflunomide	0.7	0.3	0.3	0.001
Oral CS use, %	51.8	55.4	63.2	< 0.001
Maximum dose of oral CS ^c^≥ 10 mg/day, %	22.2	20.1	22.2	0.016
Mean dose of oral CS ^c^≥ 10 mg/day, %	12.5	9.9	12.0	< 0.001
Mean dose of oral CS ^c^≥ 7.5 mg/day, %	16.0	13.4	16.7	< 0.001

*bDMARDs* Biological disease-modifying antirheumatic drugs, *CS* Corticosteroids, *csDMARDs* Conventional synthetic disease-modifying antirheumatic drugs, *JAKIs* Janus kinase inhibitors, *TNF* Tumor necrosis factor

^a^Numbers are the percentages of the patients with the prescription of the medication under the treatment with bDMARDs/JAKIs or methotrexate

^b^Fisher’s exact test

^c^Doses of oral corticosteroids were converted to prednisolone equivalent dose

During exposure to MTX, the proportion of patients who were treated with immunosuppressive DMARDs in the older elderly group was slightly smaller than those in the other groups. As observed during the exposure to bDMARDs/JAKIs, the use of oral CS was more frequent in the elderly and older elderly groups than in the young group.

### Occurrence of HI

**Fig. 2:**
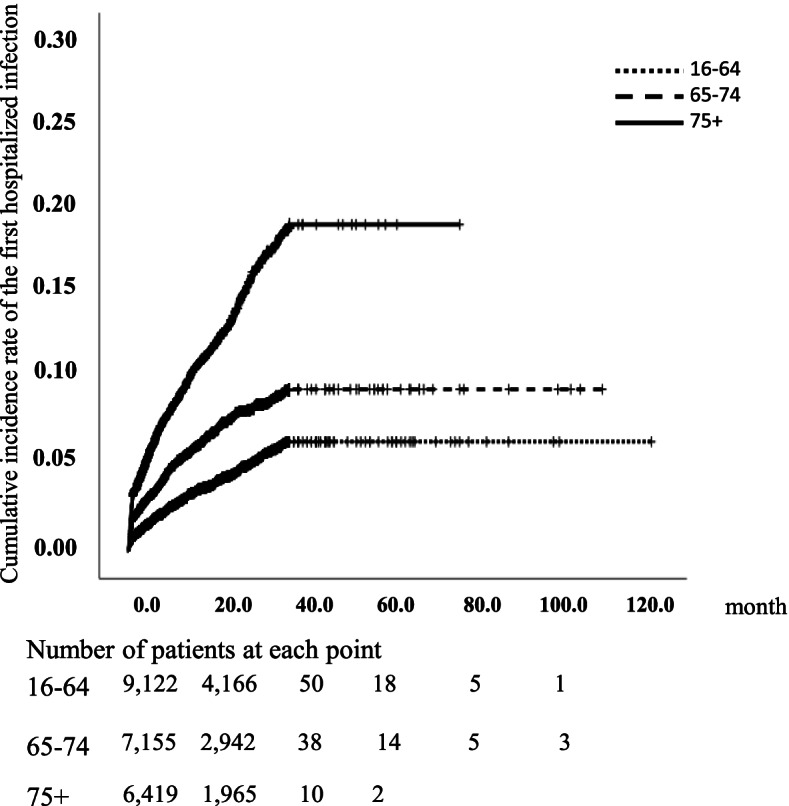
Time to the first hospitalized infection in each group. The time to the first hospitalized infection using the Kaplan–Meier method and log-rank test. The *x*-axis indicates time, and the *y*-axis indicates the cumulative incidence rate of the first hospitalized infection

During the observation period, 1811 HIs occurred in 1467 patients (359 patients in the young group, 442 patients in the elderly group, and 666 patients in the older elderly group). The IRs of HI (/100 PY, 95% confidence interval) were 3.20 [2.92–3.50] in the young group, 4.99 [4.58–5.43] in the elderly group, and 10.14 [9.45–10.85] in the older elderly group (Table 3). Significantly higher IRs were observed in the elderly and older elderly groups compared to the young group (IRR, 1.56 [1.38–1.77] for the elderly group and 3.17 [2.83–3.55] for the older elderly group). Most of the patients who developed HI had only one HI during the observation period (86.4% in all groups; 88.3% in the young group, 84.8% in the elderly group, and 86.5% in the older elderly group; *p* = 0.362 by Fisher’s exact test). Figure 2 shows the cumulative incidence rate of the first HI in each group. The median days (IQR) from the start of observation until the first HI was 164 days [24, 423] in all age groups, 236 days [53, 532] in the young group, 155 days [22, 384] in the elderly group, and 138 days [21, 403] in the older elderly group. There were significant differences in the time to the first HI among age groups (*p* < 0.001 for the elderly group versus the young group, *p* < 0.001 for the older elderly group versus the young group by log-rank test).

**Table 3:** Incidence rate per 100 patient-years and incidence rate ratio of hospitalized infection

	Young (14,668.59 PY)	Elderly (10,611.63 PY)	Older elderly (7,911.10 PY)
Number of HIs	473	534	804
Overall IR [95% CI]	3.20 [2.92–3.50]	4.99 [4.58–5.43]	10.14 [9.45–10.85]
IRR [95% CI] (vs. young)	Reference	1.56 [1.38–1.77]	3.17 [2.83–3.55]
IR [95% CI] during exposure to MTX	2.34 [2.03–2.69]	4.90 [4.39–5.46]	11.0 [10.1–12.0]
IR [95% CI] during exposure to bDMARDs/JAKIs	4.33 [3.84–4.87]	5.12 [4.47–5.86]	8.74 [7.75-–9.82]
IRR [95% CI] (bDMARDs/JAKIs vs. MTX)	1.85 [1.54–2.22]	1.05 [0.88–1.24]	0.79 [0.68–0.92]

*bDMARDs* Biological disease-modifying antirheumatic drugs, *CI* Confidence interval, *IR* Incidence rate, *IRR* Incidence rate ratio, *JAKIs* Janus kinase inhibitors, *MTX* Methotrexate, *PY* Patient-year

### Comparison of the risk of HI between TT and MTX exposures in each age group

**Fig. 3:**
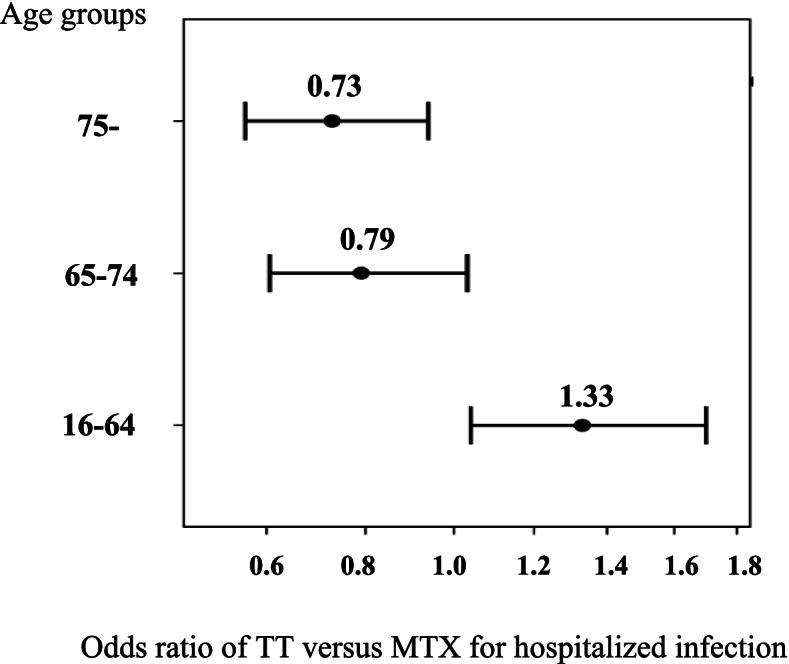
Adjusted odds ratio for hospitalized infection under targeted therapy in each group. Figure 3 shows the odds ratios (ORs) for hospitalized infection during exposure to targeted therapy (reference: exposure to MTX) with 95% confidence intervals (CIs) in each group after adjusting for age, sex, comorbidity, medications for RA at the index month, history of HI, and calendar year at the start of observation. The *x*-axis indicates age, and the *y*-axis indicates the adjusted ORs in each group. HI; hospitalized infection, MTX; methotrexate, RA; rheumatoid arthritis, TT: targeted therapy

The IRs of HI increased with age during exposure to either MTX or bDMARDs/JAKIs, but the unadjusted IRRs (TT vs. MTX) decreased with age (1.85 [1.54–2.22] in the young group, 1.05 [0.88–1.24] in the elderly group, and 0.79 [0.68–0.92] in the older elderly group). To investigate the associations between the exposure to bDMARDs/JAKIs and HI more precisely, we calculated the adjusted OR of bDMARDs/JAKIs versus MTX exposure for HI. After adjusting for patients’ characteristics and medications for RA using a logistic regression model as described in the “Methods” section, the OR of bDMARDs/JAKIs exposure in each group was 1.33 (1.04–1.70, *p* = 0.021) in the young group, 0.79 (0.61–1.03, *p* = 0.084) in the elderly group, and 0.73 (0.56–0.94, *p* = 0.015) in the older elderly group (Fig. 3). Odds ratios of patients’ characteristics other than medication in each group are presented in the Supplementary Table 2. The common risk factors among groups were presence of chronic pulmonary disease, and history of hospitalized infection. Among the bDMARDs users (i.e., excluding JAKIs users from bDMARDs/JAKIs users as described in the “Methods” section) (*n* = 8877 in the young group, *n* = 6949 in the elderly group, *n* = 6249 in the older elderly group), the ORs of bDMARDs versus MTX exposure for HI in each group were similar to the ORs of bDMARDs/JAKIs exposure (1.36 [1.06–1.74], *p* = 0.015 in the young group, 0.81 [0.61–1.06], *p* = 0.119 in the elderly group, 0.69 [0.53–0.91], *p* = 0.008 in the older elderly group) (Supplementary Fig. 2).

## Discussion

In this study, using a large health insurance database, we conducted an on-drug analysis of HI in patients with RA and showed that the absolute risk for HI in patients with RA during exposure to MTX or bDMARD/JAKI increased with age, but that the relative risk for HI during exposure to bDMARDs/JAKIs compared to MTX decreased with age from 1.33 (95% CI 1.04–1.70) in the young group to 0.73 (0.56–0.94) in the older elderly group.

The IRs of HI in the elderly and older elderly groups were almost consistent with those in previous studies, although some methods, such as the definition of infections and the observation period, were different across studies. Previous studies reported that the IR of serious infection under treatment with bDMARDs in elderly patients with RA (≥65 years old) was 4–8 per 100 PY [8, 16, 24, 25]. Only one study showed that the IR of serious infection in patients who were 75 years or older and received TNF inhibitors was 8.3/100 PY [25]. To date, few studies have investigated the IR of infection in older elderly patients with RA. It is clinically important to show the risk of infection in these populations, considering the rapidly increasing number of older elderly patients in aging and super-aging societies around the world.

In this study, exposure to TT did not increase the risk of HI in elderly and older elderly groups versus methotrexate; these findings are supported by those of previous studies [24, 26]. American large claims data including a population aged 65 years or older showed that TNF inhibitor use was not a significant risk factor for serious bacterial infection [26]. A Japanese retrospective study also reported that there were no significant associations between bDMARD use and serious infections in elderly patients with RA [24]. The decreased risk for HI under TT versus MTX exposure in the elderly and older elderly groups can be explained as follows. First, treatments in these groups might be modified to prevent infection by attending physicians, including the choice of bDMARDs and less use of MTX. Significantly more patients were treated with TNF inhibitors in the young group than in the elderly and older elderly groups whereas the proportion of patients who were treated with abatacept significantly increased with age (Table 2). It has been reported that the use of TNF inhibitors leads to a 20–40% increased risk of HI compared to abatacept use in patients aged 65 years or older [22]. A Japanese observational study also showed that approximately half of patients aged 75 years or older used abatacept, which led to comparable retention rates of bDMARDs between patients aged 65–74 years and ≥75 years [21]. Moreover, it is likely that the attending physicians attempted to minimize the risk of adverse events of MTX considering impaired renal and/or liver function in elderly and older elderly groups. Another reason for the decreased risk of TT in elderly and older elderly groups might be the recent improvement in the management of bDMARD therapy. Kojima et al. reported that the cumulative incidence of adverse events, including infections, was significantly lower in the later calendar year of the initiation of bDMARDs compared with the early calendar year (≤ 2005) [27]. The increased number of available bDMARDs in the patients who initiated treatment in later calendar years could explain the lower incidence of adverse events because of appropriate selection of an agent considering patients’ benefit–risk balance and better disease control. In addition, a series of pharmacoepidemiological studies on the safety of bDMARDs worldwide have provided the risk for and risk factors of infection in patients with RA, which has led to improved risk management under TT. Since this study population started TT between 2008 and 2018, appropriate selection of patients for TT and risk management during treatment with TT could be performed. Other possible reasons of the decreased risk of TT in the elderly and older elderly groups might be that preventive measures of infections such as vaccination and prophylaxis were performed more frequently than in the young group because physicians considered them as a high-risk population of infections. However, information of vaccination as well as laboratory and imaging data relevant to the risk of infections are not available in the claims data.

The use of oral CS is also an important risk factor for infections in patients with RA [24, 26]. In this study, a higher proportion of patients who were treated with oral CS was observed in the elderly and older elderly groups than in the young group, and the adjusted ORs of oral CS use for HI versus no oral CS use in each age group were approximately 2.0, which were significantly elevated (data not shown). In addition, we confirmed that ORs of oral CS after categorization by mean dose (>0 and <7.5 mg/day, ≥7.5 mg/day) versus no use were significantly increased in each age group (data not shown). As it is necessary to suppress inflammation in the early course of treatment for RA, the use of CS for a short period of time is recommended when initiating or changing csDMARDs [28]. It is important to use oral CS at the lowest dose for the shortest period.

Patients’ characteristics such as comorbidity affected the risk of HI. In this study, the common risk factors across the age groups were presence of chronic pulmonary disease and history of hospitalized infection (Supplementary Table 2). Thus, patients with these risk factors have a high risk of HI, and it is important to consider the appropriate risk management of infection to prevent HI.

This study has some limitations. First, there is a possibility of the misclassification of RA cases although the validation study for the definition of RA has been already performed [20]. To exclude the false RA cases as much as possible, we defined using not only ICD10 codes but the prescription of MTX, bDMARDs, or JAKIs. Second, we could not distinguish elderly onset RA cases from younger onset elderly patients considering that it is impossible to define the correct disease duration of RA in the claims data due to the lack of clinical information. Third, we must consider residual confounding because we could not adjust for patients’ characteristics such as RA disease activity and physical function due to the lack of clinical data. Patients with high disease activity or poor physical function have a higher risk of serious infection than those without [7, 29]. Forth, there is a possibility that HI was overestimated since the definition of HI has not yet been validated in the claims data. Fifth, we did not investigate the differences in dosages of bDMARDs/JAKIs across the age groups because it was difficult to accurately identify them in the claims data. In addition, we could not estimate the risk of each bDMARDs/JAKIs due to the small number of the patients treated with each drug. Further studies using a larger number of patients than that of the present study are needed.

## Conclusions

The overall incidence of HI was higher in both elderly and older elderly patients compared to young patients, whereas we found that the risks of HI in both elderly and older elderly patients exposed to TT versus MTX were not significantly increased compared to young patients. These results suggest that TT can be provided safely to elderly and older elderly patients with RA with careful risk management and appropriate adjustment for treatments and that treatment strategy may differ across the age groups.

## Supplementary Information

**Additional file 1: Supplementary Figure 1.** Follow-up. The black column indicates exposure to bDMARDs/JAKI or MTX. The white and striped arrows indicate the observation period for MTX and bDMARDs/JAKIs, respectively. The last day of exposure to MTX or JAKI was defined as the last day of a prescription for MTX or JAKI, respectively, plus supply days and 30 days as a grace period. a) When a patient received bDMARDs/JAKIs without MTX, the observation period was from the index month to the last exposure to bDMARDs/JAKIs. The observation period was similar to that of the bDMARDs/JAKIs. b) When a patient received MTX without bDMARDs/JAKIs, the observation period was from the index month to the last exposure to MTX. The observation period was similar to that for MTX. c) When a patient received bDMARDs/JAKIs and MTX concomitantly, MTX was discontinued and the observation period was from the index month to the last exposure to bDMARDs/JAKIs. The observation period was similar to that of the bDMARDs/JAKIs. d) When a patient received bDMARDs/JAKIs and MTX concomitantly, bDMARDs/JAKIs were stopped and the observation period was from the index month to the last exposure to bDMARDs/JAKIs. The observation period was similar to that of the bDMARDs/JAKIs. e) When a patient received MTX, bDMARDs/JAKIs were added, and the observation period was from the index month to the last exposure to bDMARDs/JAKIs. The observation period contributed to either MTX or bDMARD/JAKIs, based on the exposure to each drug. f) When a patient received bDMARDs/JAKIs with MTX, the observation period was from the index month to the last exposure to bDMARDs/JAKIs. The observation period was similar to that of the bDMARDs/JAKIs. g) When a patient received MTX and switched to bDMARDs/JAKIs, the observation period contributed to either MTX or bDMARDs/JAKIs based on the exposure to each drug. h) When a patient received bDMARDs/JAKIs and switched to MTX, the observation period was from the index month to the last exposure to bDMARDs/JAKIs. The observation period was similar to that of the bDMARDs/JAKIs. **Supplementary Figure 2.** Adjusted odds ratio for hospitalized infection during exposure to biological DMARDs in each group. Supplementary Figure 2 shows the odds ratios (ORs) for hospitalized infection during exposure to biological DMARD (reference: exposure to MTX) with 95% confidence intervals (CIs) in each group after adjusting for age, sex, comorbidity, medications for RA at the index month, history of HI, and calendar year at the start of observation. The x-axis indicates age, and the y-axis indicates the adjusted ORs in each group. HI; hospitalized infection, MTX; methotrexate, RA; rheumatoid arthritis, bDMARDs: biological disease-modifying antirheumatic drugs.

**Additional file 2: Supplementary Table 1.** ICD-10 codes and medications for the definition of hospitalized infection. **Supplementary Table 2.** The odds ratios of patients’ characteristics other than medications in the multivariable analysis in each age group.

## Data Availability

The datasets generated during and/or analyzed during the current study are not publicly available due to their proprietary nature and the associated restrictions that apply to their availability to external sources.

## Abbreviations

bDMARDs: Biological disease-modifying antirheumatic drugs
CS: Corticosteroid
DM: Diabetes mellitus
DPC/PDPS: The Diagnostic Procedure Combination/per diem payment system
HI: Hospitalized infection
IR: Incidence rate
IRR: Incidence rate ratio
ICD-10: The International Statistical Classification of Diseases and Related Health Problems, 10th Revision
JAKIs: Janus kinase inhibitors
MDV: The Medical Data Vision
MTX: Methotrexate
OR: Odds ratio
PY: Patient-years
RA: Rheumatoid arthritis
TT: Targeted therapy

## References

[1] Widdifield J, Paterson JM, Bernatsky S (2014). The epidemiology of rheumatoid arthritis in Ontario, Canada. Arthritis Rheumatol.

[2] Kaipiainen-Seppanen O, Kautiainen H (2006). Declining trend in the incidence of rheumatoid factor-positive rheumatoid arthritis in Finland 1980-2000. J Rheumatol.

[3] Myasoedova E, Crowson CS, Kremers HM, Therneau TM, Gabriel SE (2010). Is the incidence of rheumatoid arthritis rising?: results from Olmsted County, Minnesota, 1955-2007. Arthritis Rheum.

[4] Crowson CS, Matteson EL, Myasoedova E (2011). The lifetime risk of adult-onset rheumatoid arthritis and other inflammatory autoimmune rheumatic diseases. Arthritis Rheum.

[5] Nakajima A, Sakai R, Inoue E, Harigai M (2020). Prevalence of patients with rheumatoid arthritis and age-stratified trends in clinical characteristics and treatment, based on the National Database of Health Insurance Claims and Specific Health Checkups of Japan. Int J Rheum Dis.

[6] Tan TC, Gao X, Thong BY (2017). Comparison of elderly- and young-onset rheumatoid arthritis in an Asian cohort. Int J Rheum Dis.

[7] Sakai R, Komano Y, Tanaka M (2012). Time-dependent increased risk for serious infection from continuous use of tumor necrosis factor antagonists over three years in patients with rheumatoid arthritis. Arthritis Care Res.

[8] Widdifield J, Bernatsky S, Paterson JM (2013). Serious infections in a population-based cohort of 86,039 seniors with rheumatoid arthritis. Arthritis Care Res.

[9] Wolfe F, Caplan L, Michaud K (2006). Treatment for rheumatoid arthritis and the risk of hospitalization for pneumonia: associations with prednisone, disease-modifying antirheumatic drugs, and anti-tumor necrosis factor therapy. Arthritis Rheum.

[10] Doran MF, Crowson CS, Pond GR, O'Fallon WM, Gabriel SE (2002). Predictors of infection in rheumatoid arthritis. Arthritis Rheum.

[11] Greenberg JD, Reed G, Kremer JM (2010). Association of methotrexate and tumour necrosis factor antagonists with risk of infectious outcomes including opportunistic infections in the CORRONA registry. Ann Rheum Dis.

[12] Curtis JR, Patkar N, Xie A (2007). Risk of serious bacterial infections among rheumatoid arthritis patients exposed to tumor necrosis factor alpha antagonists. Arthritis Rheum.

[13] Strangfeld A, Eveslage M, Schneider M (2011). Treatment benefit or survival of the fittest: what drives the time-dependent decrease in serious infection rates under TNF inhibition and what does this imply for the individual patient?. Ann Rheum Dis.

[14] Bechman K, Subesinghe S, Norton S (2019). A systematic review and meta-analysis of infection risk with small molecule JAK inhibitors in rheumatoid arthritis. Rheumatology (Oxford).

[15] Bechman K, Oke A, Yates M (2020). Is background methotrexate advantageous in extending TNF inhibitor drug survival in elderly patients with rheumatoid arthritis? An analysis of the British Society for Rheumatology Biologics Register. Rheumatology (Oxford, England).

[16] Leon L, Gomez A, Vadillo C (2018). Severe adverse drug reactions to biological disease-modifying anti-rheumatic drugs in elderly patients with rheumatoid arthritis in clinical practice. Clin Exp Rheumatol.

[17] Dalal DS, Duran J, Brar T (2019). Efficacy and safety of biological agents in the older rheumatoid arthritis patients compared to young: a systematic review and meta-analysis. Semin Arthritis Rheum.

[18] Sakai R, Tanaka E, Nishina H, Suzuki M, Yamanaka H, Harigai M (2019). Risk of opportunistic infections in patients with antineutrophil cytoplasmic antibody-associated vasculitis, using a Japanese health insurance database. Int J Rheum Dis.

[19] Sakai R, Honda S, Tanaka E (2020). The risk of hospitalized infection in patients with systemic lupus erythematosus treated with hydroxychloroquine. Lupus..

[20] Kubota K, Yoshizawa M, Takahashi S, Fujimura Y, Nomura H, Kohsaka H (2021). The validity of the claims-based definition of rheumatoid arthritis evaluated in 64 hospitals in Japan. BMC Musculoskelet Disord.

[21] Murota A, Kaneko Y, Yamaoka K, Takeuchi T (2016). Safety of biologic agents in elderly patients with rheumatoid arthritis. J Rheumatol.

[22] Yun H, Xie F, Delzell E (2016). Comparative risk of hospitalized infection associated with biologic agents in rheumatoid arthritis patients enrolled in Medicare. Arthritis Rheumatol.

[23] Quan H, Sundararajan V, Halfon P (2005). Coding algorithms for defining comorbidities in ICD-9-CM and ICD-10 administrative data. Med Care.

[24] Kawashima H, Kagami SI, Kashiwakuma D (2017). Long-term use of biologic agents does not increase the risk of serious infections in elderly patients with rheumatoid arthritis. Rheumatol Int.

[25] Galloway JB, Hyrich KL, Mercer LK (2011). Anti-TNF therapy is associated with an increased risk of serious infections in patients with rheumatoid arthritis especially in the first 6 months of treatment: updated results from the British Society for Rheumatology Biologics Register with special emphasis on risks in the elderly. Rheumatology (Oxford).

[26] Schneeweiss S, Setoguchi S, Weinblatt ME (2007). Anti-tumor necrosis factor alpha therapy and the risk of serious bacterial infections in elderly patients with rheumatoid arthritis. Arthritis Rheum.

[27] Kojima T, Takahashi N, Funahashi K (2016). Improved safety of biologic therapy for rheumatoid arthritis over the 8-year period since implementation in Japan: long-term results from a multicenter observational cohort study. Clin Rheumatol.

[28] Smolen JS, Landewe RBM, Bijlsma JWJ (2020). EULAR recommendations for the management of rheumatoid arthritis with synthetic and biological disease-modifying antirheumatic drugs: 2019 update. Ann Rheum Dis.

[29] Au K, Reed G, Curtis JR (2011). High disease activity is associated with an increased risk of infection in patients with rheumatoid arthritis. Ann Rheum Dis.

