# Supplementary material for: Unincreased risk of hospitalized infection under targeted therapies versus methotrexate in elderly patients with rheumatoid arthritis: a retrospective cohort study
Source: Arthritis Res Ther. 2022 Jun 10;24:135. doi: 10.1186/s13075-022-02807-9 (PMC9185865; doi:10.1186/s13075-022-02807-9)
Supplement: Supplementary file 1 — Additional file 1: Supplementary Figure 1. Follow-up. The black column indicates exposure to bDMARDs/JAKI or MTX. The white and striped arrows indicate the observation period for MTX and bDMARDs/JAKIs, respectively. The last day of exposure to MTX or JAKI was defined as the last day of a prescription for MTX or JAKI, respectively, plus supply days and 30 days as a grace period. a) When a patient received bDMARDs/JAKIs without MTX, the observation period was from the index month to the last exposure to bDMARDs/JAKIs. The observation period was similar to that of the bDMARDs/JAKIs. b) When a patient received MTX without bDMARDs/JAKIs, the observation period was from the index month to the last exposure to MTX. The observation period was similar to that for MTX. c) When a patient received bDMARDs/JAKIs and MTX concomitantly, MTX was discontinued and the observation period was from the index month to the last exposure to bDMARDs/JAKIs. The observation period was similar to that of the bDMARDs/JAKIs. d) When a patient received bDMARDs/JAKIs and MTX concomitantly, bDMARDs/JAKIs were stopped and the observation period was from the index month to the last exposure to bDMARDs/JAKIs. The observation period was similar to that of the bDMARDs/JAKIs. e) When a patient received MTX, bDMARDs/JAKIs were added, and the observation period was from the index month to the last exposure to bDMARDs/JAKIs. The observation period contributed to either MTX or bDMARD/JAKIs, based on the exposure to each drug. f) When a patient received bDMARDs/JAKIs with MTX, the observation period was from the index month to the last exposure to bDMARDs/JAKIs. The observation period was similar to that of the bDMARDs/JAKIs. g) When a patient received MTX and switched to bDMARDs/JAKIs, the observation period contributed to either MTX or bDMARDs/JAKIs based on the exposure to each drug. h) When a patient received bDMARDs/JAKIs and switched to MTX, the observation period was from the index month to the last ex [file 13075_2022_2807_MOESM1_ESM.pptx]

## Slide 1
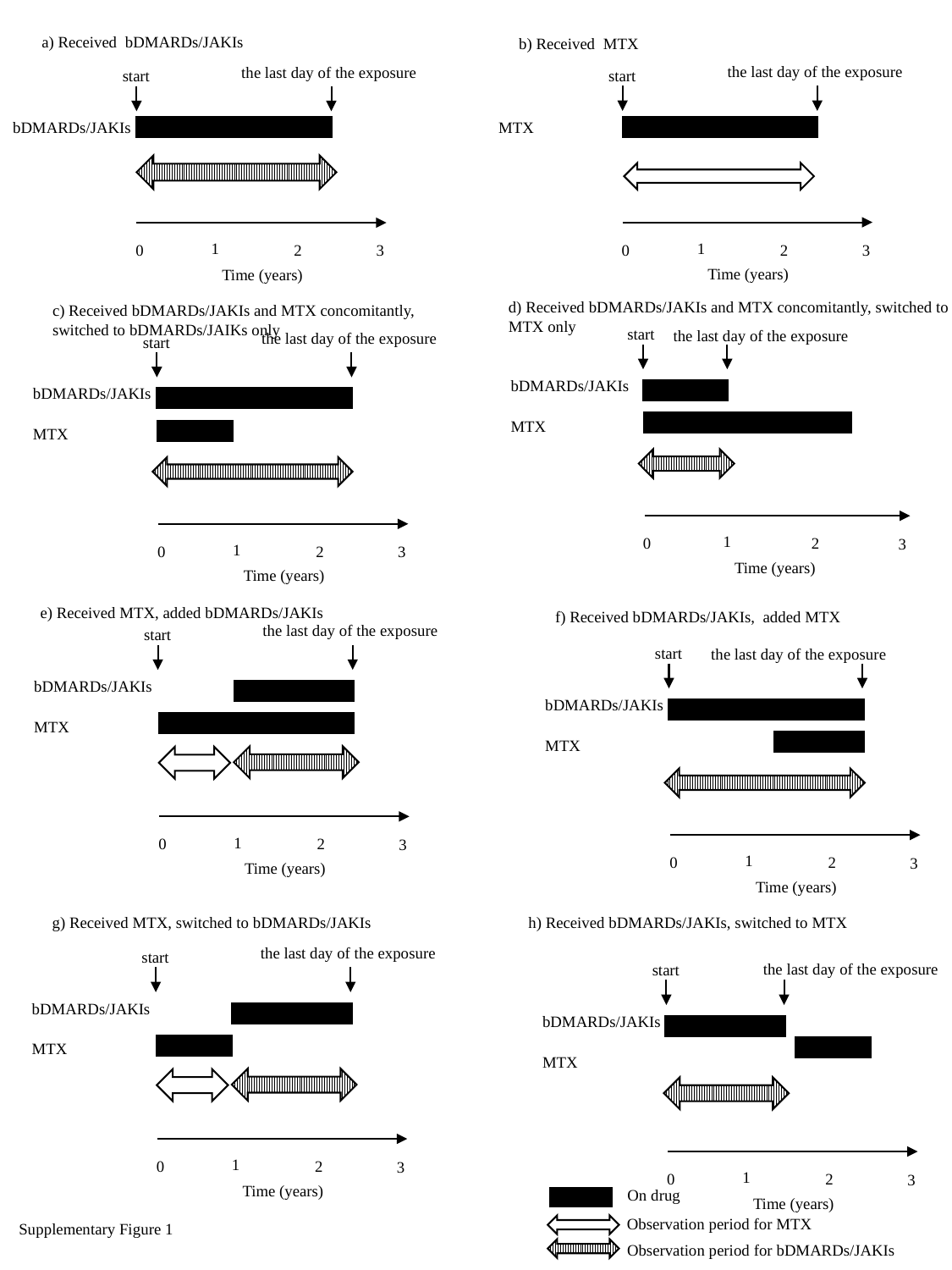

a) Received bDMARDs/JAKIs
b) Received MTX
the last day of the exposure
start
MTX
1
0
2
3
Time (years)
the last day of the exposure
start
bDMARDs/JAKIs
1
0
2
3
Time (years)
d) Received bDMARDs/JAKIs and MTX concomitantly, switched to MTX only
start
the last day of the exposure
bDMARDs/JAKIs
MTX
1
0
2
3
Time (years)
c) Received bDMARDs/JAKIs and MTX concomitantly, switched to bDMARDs/JAIKs only
the last day of the exposure
start
bDMARDs/JAKIs
MTX
1
0
2
3
Time (years)
e) Received MTX, added bDMARDs/JAKIs
the last day of the exposure
start
bDMARDs/JAKIs
MTX
1
0
2
3
Time (years)
f) Received bDMARDs/JAKIs, added MTX
start
the last day of the exposure
bDMARDs/JAKIs
MTX
1
0
2
3
Time (years)
g) Received MTX, switched to bDMARDs/JAKIs
the last day of the exposure
start
bDMARDs/JAKIs
MTX
1
0
2
3
Time (years)
h) Received bDMARDs/JAKIs, switched to MTX
the last day of the exposure
start
bDMARDs/JAKIs
MTX
1
0
2
3
Time (years)
On drug
Observation period for MTX
Observation period for bDMARDs/JAKIs
Supplementary Figure 1

## Slide 2
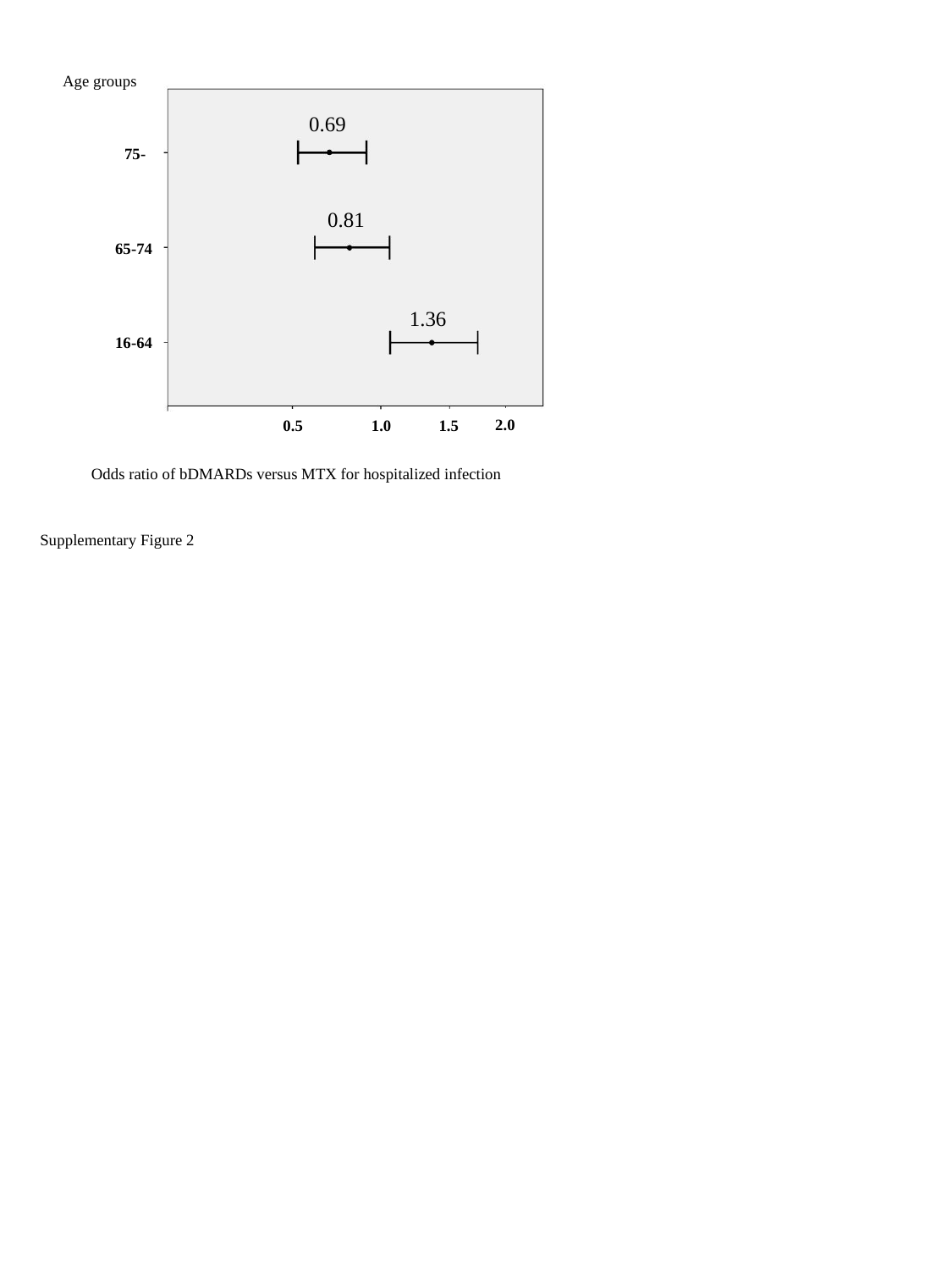

Age groups
75-
65-74
16-64
2.0
1.5
0.5
1.0
0.69
0.81
1.36
Odds ratio of bDMARDs versus MTX for hospitalized infection
Supplementary Figure 2
