# Supplementary material for: Unincreased risk of hospitalized infection under targeted therapies versus methotrexate in elderly patients with rheumatoid arthritis: a retrospective cohort study
Source: Arthritis Res Ther. 2022 Jun 10;24:135. doi: 10.1186/s13075-022-02807-9 (PMC9185865; doi:10.1186/s13075-022-02807-9)
Supplement: Supplementary file 2 — Additional file 2: Supplementary Table 1. ICD-10 codes and medications for the definition of hospitalized infection. Supplementary Table 2. The odds ratios of patients’ characteristics other than medications in the multivariable analysis in each age group. [file 13075_2022_2807_MOESM2_ESM.docx]

The cover page of supplementary materials

Title:

# Decreased risk of hospitalized infection under targeted therapies in older elderly Japanese patients with rheumatoid arthritis: a retrospective study

Authors: Ryoko Sakai^1,2^, Eiichi Tanaka^1^, Masako Majima^1^, Masayoshi Harigai^1^

Affiliations:

^1^ Division of Rheumatology, Department of Internal Medicine, Tokyo Women’s Medical University School of Medicine, 8-1, Kawada-cho, Sinjuku-ku, Tokyo 162–8666, Japan

^2^Division of Multidisciplinary Management of Rheumatic Diseases, Department of Rheumatology, Tokyo Women’s Medical University School of Medicine, 8-1, Kawada-cho, Sinjuku-ku, Tokyo 162–8666, Japan

Corresponding author and email:

Masayoshi Harigai,

E-mail: harigai.masayoshi@twmu.ac.jp

Supplementary Table 1. ICD-10 codes and medications for the definition of hospitalized infection

| ICD-10 code | Medications |
| --- | --- |
| A00x | NA |
| A01x | NA |
| A02x | NA |
| A03x | NA |
| A040-044 | Ciprofloxacin (po) or levofloxacin (po) or sulfamethoxazole/trimethoprim (po) |
| A045 | Erythromycin (po) or ciprofloxacin (po) or doxycycline (po) |
| A047 | Metronidazole (po) or vancomycin (po) |
| A048 | Arbekacin (iv) or , vancomycin(iv, po), teicoplanin (iv) or  daptomycin (iv) or linezolid (iv) for MRSA, antibacterial drug  (iv) for others |
| A049 | Ceftriaxone (iv) or cefotaxime (iv) or ciprofloxacin (po) or levofloxacin (po) or metronidazole (po) |
| A05.x | NA |
| A06.x | Metronidazole (po) or paramomycin (po) |
| A07.x | Metronidazole (po) or tinidazole (po) |
| A08.x | NA |
| A15.x–19.x | Rifampicin (po) and isoniazid (iv, po) |
| A20.x–28.x | Antibacterial drug (iv) |
| A30.x, 32.x -49.x | Antibacterial drug (iv) |
| A31.x | (Clarithromycin and rifampicin and ethambutol) and/or streptomycin (iv) and/or kanamycin |
| A50.x–64.x | Antibacterial drug (iv) |
| A65.x–69.x | Antibacterial drug (iv) |
| A70.x–74.x | Antibacterial drug (iv) |
| A75.x–79.x | Antibacterial drug (iv) |
| A80.x–89.x | NA |
| A90.x–99.x | NA |
| B01.x | Antiviral drug (iv, po) |
| B02.x | Antiviral drug (iv, po) |
| B16.x | At least one medication for hepatitis B |
| B17.x | At least one medication for hepatitis C |
| B20.x–24.x | At least one medication for human immunodeficiency virus |
| B25.x | At least one medication for cytomegalovirus |
| B27.x | NA |
| B37.x | At least one antifungal drug for candidiasis |
| B44.x | At least one antifungal drug for aspergillosis |
| B45.x | At least one antifungal drug for cryptococcosis |
| B46.x | At least one antifungal drug for mucormycosis |
| B47.x | NA |
| B48.x–49.x | Antifungal drug (iv, po) |
| B50.x–54.x | Antimalarial drug |
| B58.x | Sulfadiazine or pyrimethomine |
| B59.x | Sulfamethoxazole/trimethoprim (po) |
| B60.x–64.x | NA |
| B65.x | Praziquantel |
| B66.x–77.x | NA |
| B78.x | Ivermectin |
| B79.x | Mebendazole |
| B80.x | NA |
| B82.x–85.x | NA |
| B86.x | Phenothrin or ivermectin |
| B87.x | NA |
| G00.x–01.x | Antibacterial drug (iv) |
| G02.x | Antibacterial drug (iv) or antifungal drug (iv, po) or antiviral drug (iv) |
| G039 | Antibacterial drug (iv) or antifungal drug (iv, po) or antiviral drug (iv) |
| G042 | Antibacterial drug (iv) |
| G048, 049 | Antibacterial drug (iv) or antifungal drug (iv, po) or antiviral drug (iv) |
| H03.0 | Antiparasitic drug |
| H13.0 | Antiparasitic drug |
| H19.2 | Antiparasitic drug |
| H22.0 | Antiparasitic drug |
| H66.x | Antibacterial drug (iv) |
| I301 | Antibacterial drug (iv) or antifungal drug (iv, po) or antiviral drug (iv) |
| I32.x | Antibacterial drug (iv) |
| I40.0 | Antiviral drug (iv) |
| I41.0–41.2 | Antibacterial drug (iv) or antifungal drug (iv, po) or antiviral drug (iv) |
| I43.0 | Antibacterial drug (iv) or antifungal drug (iv, po) or antiviral drug (iv) |
| J13.x–16.x | Antibacterial drug (iv) |
| J17.0–17.3 | Antibacterial drug (iv) or antifungal drug (iv, po) or antiviral drug (iv) |
| J18.x | Antibacterial drug (iv) or antifungal drug (iv, po) or antiviral drug (iv) |
| J20.x | Antibacterial drug (iv) or antifungal drug (iv, po) or antiviral drug (iv) |
| J21.x | Antibacterial drug (iv) or antifungal drug (iv, po) or antiviral drug (iv) |
| J36.x | Antibacterial drug (iv) |
| J85.x–86.x | Antibacterial drug (iv) or antifungal drug (iv, po) or antiviral drug (iv) |
| K35.x | Antibacterial drug (iv) |
| K81.x | Antibacterial drug (iv) |
| L02.x–03.x | Antibacterial drug (iv) |
| L04.. | NA |
| L08.x | Antibacterial drug (iv) or antifungal drug (iv, po) or antiviral drug (iv) |
| M00.x | Antibacterial drug (iv) |
| M726 | Antibacterial drug (iv) |
| M86 | Antibacterial drug (iv) |
| N390 | Antibacterial drug (iv) |
| N73 | Antibacterial drug (iv) |
| O23 | Antibacterial drug (iv) |
| O85.x–86.x | Antibacterial drug (iv) |
| O910, 911 | Antibacterial drug (iv) |
| O98.x | Antibacterial drug (iv) |

NA, not applicable

NA of medications means that infections were defined by ICD-10 codes only.

Supplementary Table 2. The odds ratios of patients’ characteristics other than medications in the multivariable analysis in each age group

1. **Young group (n=9,122)**

| **Covariate** | **Odds ratio [95% CI]** | **P value** |
| --- | --- | --- |
| Age by decade | 1.42 [1.26-1.60] | <0.001 |
| Male | 1.03 [0.81-1.32] | 0.800 |
| Diabetes mellitus | 1.48 [1.04-2.09] | 0.028 |
| Chronic pulmonary disease | 1.99 [1.54-2.58] | <0.001 |
| Renal disease | 1.32 [0.72-2.40] | 0.366 |
| Congestive Heart failure | 1.48 [1.02-2.15] | 0.042 |
| History of HI | 3.03 [2.06-4.45] | <0.001 |

HI, hospitalized infection

1. **Elderly group (n=7,155)**

| **Covariate** | **Odds ratio [95% CI]** | **P value** |
| --- | --- | --- |
| Age by decade | 1.26 [0.90-1.77] | 0.186 |
| Male | 0.81 [0.66-1.00] | 0.049 |
| Diabetes mellitus | 1.25 [0.96-1.64] | 0.099 |
| Chronic pulmonary disease | 1.63 [1.29-2.05] | <0.001 |
| Renal disease | 0.97 [0.60-1.56] | 0.894 |
| Congestive Heart failure | 1.69 [1.31-2.19] | <0.001 |
| History of HI | 2.61 [1.88-3.64] | <0.001 |

HI, hospitalized infection

1. **Older elderly group (n=6,419)**

| **Covariate** | **Odds ratio [95% CI]** | **P value** |
| --- | --- | --- |
| Age by decade | 1.69 [1.41-2.06] | <0.001 |
| Male | 0.73 [0.61-0.87] | <0.001 |
| Diabetes mellitus | 0.95 [0.74-1.23] | 0.691 |
| Chronic pulmonary disease | 1.67 [1.38-2.04] | <0.001 |
| Renal disease | 0.65 [0.43-0.99] | 0.045 |
| Congestive Heart failure | 1.20 [0.97-1.47] | 0.095 |
| History of HI | 1.82 [1.38-2.39] | <0.001 |

HI, hospitalized infection
